# Targeting the PI3K/STAT3 axis modulates age‐related differences in macrophage phenotype in rats with myocardial infarction

**DOI:** 10.1111/jcmm.14526

**Published:** 2019-07-17

**Authors:** Chih‐Chan Lin, Syue‐yi Chen, Hsiao‐Yin Lien, Shinn‐Zong Lin, Tsung‐Ming Lee

**Affiliations:** ^1^ Department of Medical Research Chi‐Mei Medical Center Tainan Taiwan; ^2^ Cardiovascular Institute, An Nan Hospital, China Medical University Tainan Taiwan; ^3^ Department of pharmacy Kaohsiung Veterans general hospital Tainan branch Tainan Taiwan; ^4^ Bioinnovation Center, Tzu Chi foundation Department of Neurosurgery Buddhist Tzu Chi General Hospital, Tzu Chi University Yunlin Taiwan; ^5^ Cardiovascular Institute, An Nan Hospital Tainan Taiwan; ^6^ Department of Medicine China Medical University Taichung Taiwan

**Keywords:** ageing, fibrosis, *n*‐butylidenephthalide, phosphatidyl‐inositol‐3‐kinase, signal transducer and activator of transcription 3

## Abstract

Ageing is associated with impaired repair mechanisms in cardiovascular diseases. Macrophages contribute to cardiac fibrosis after myocardial infarction (MI). The phosphatidyl‐inositol‐3‐kinase (PI3K) pathway has been shown to play a role in cardiac remodelling after MI. It remained unclear whether *n*‐butylidenephthalide, a major component of *Angelica sinensis,* can attenuate cardiac fibrosis by regulating the PI3K/signal transducer and activator of transcription 3 (STAT3)‐mediated macrophage phenotypes in ageing rats after MI. Twenty‐four hours after ligation of the left anterior descending artery, young (2‐month‐old) and ageing (18‐month‐old) male Wistar rats were treated with either vehicle or *n*‐butylidenephthalide for 4 weeks. There were similar infarct sizes in both age groups. Compared with young rats, ageing rats exhibited significant increased cardiac fibrosis after MI, which can be attenuated after administering *n*‐butylidenephthalide. MI was associated with decreased activities of PI3K and STAT3 in ageing rats compared with young rats. In both age groups, *n*‐butylidenephthalide effectively provided a significant increase of STAT3 phosphorylation, STAT3 activity, STAT3 nuclear translocation, myocardial IL‐10 levels and the percentage of M2c macrophage and a decrease of myofibroblast infiltration. The effects of *n*‐butylidenephthalide on increased IL‐10 levels were reversed by LY294002 or S3I‐201. Furthermore, LY294002 abolished the STAT3 phosphorylation, whereas PI3K activity was not affected following the inhibition of STAT3. In conclusions, the host environment is responsible for ageing‐related myofibroblast dysregulation in response to MI which can be improved by administering *n*‐butylidenephthalide via macrophage differentiation towards M2 phenotype by targeting the PI3K/STAT3 axis.

## 1 INTRODUCTION

Cardiac remodelling after myocardial infarction (MI) is clinically characterized by a complex inflammation process. Among the cells involved in mediating inflammation, macrophages act as major effector cells in tissue repair and fibrosis.^1^ Macrophages can be categorized as M1 and M2 macrophages at the opposing ends. M1 macrophages are often thought of as pro‐inflammatory action and express inflammatory genes, including iNOS. In contrast, M2 macrophages are involved in the resolution of inflammation 2 and can secrete arginase‐1 and IL‐10.^3^ Excessive activation of M1 macrophages and delayed transition from M1 to M2 worsen post‐MI remodelling.^4^ Despite these recent advances, the signalling pathways by skewing M1 macrophages to the M2 Phenotype are poorly understood. The signal transducer and activator of transcription 3 (STAT3) signalling pathway has been shown to be a key player regulating macrophage phenotype. STAT3 belongs to the STAT family of transcription factors that drives macrophage polarization to M2c phenotype. Very recently, sustained phosphatidylinositol 3‐kinase (PI3K) activation has been shown to increase lung fibrosis by modulating the macrophage polarization.^5^ Although PI3K and STAT3 proteins represent two distinct cellular regulatory systems, a functional link between these two systems has been shown by the observation that a specific inhibitor of PI3K reduces the level of STAT3 phosphorylation.^6^ Thus, it is assumed that PI3K can tune macrophage phenotype at least in part by STAT3 translocation; however, the exact role of PI3K/STAT3 signalling on macrophage skewing remained unknown.

Ageing has been shown to associate with a chronic, low‐grade inflammation.^7^ Ageing may modulate M1/M2 activation and polarization.^8^, ^9^ Impaired macrophage polarization in the elderly dysregulates the development of the host response, anticipating age‐related increases in cardiac susceptibility.^10^ Age‐dependent macrophage changes lead to functional deficits or impaired cardiac injury responses. The resolution of acute inflammation is an actively regulated mechanism governed by which anti‐inflammatory signals suppress inflammation, clear necrotic cells and promote healing, facilitating the recovery of normal tissue function. Dendritic cells exhibited age‐related phagocytotic dysfunction with an impaired signalling of PI3K/AKT pathway.^11^ A failure to activate STAT3 in aged keratinocytes leads to impaired re‐epithelization process following skin wounding.^12^ The disruptions in the signalling cascades may in part provide the mechanism underlying an increased propensity for higher inflammation that couples the ageing process. Whether macrophage subsets can be manipulated to improve cardiac repair after injury remains to be explored.

Butylidenephthalide is an active compound of *Angelica sinensis*.^13^ *Angelica sinensis*, a traditional Chinese herbal medicine, has been used for the treatment of various health problems including cardiovascular diseases.^14^ Butylidenephthalide has been reported to improve cognitive impairment via the activation of PI3K.^15^ The alterations of the ageing heart on macrophage phenotypes may compromise ventricular remodelling after MI. However, whether the effect of *n*‐butylidenephthalide on cardiac fibrosis by activating PI3K signalling after infarction remained unclear. In this study, we assessed (a) whether there were age‐related changes in macrophage phenotype and the potential impact of these changes on MI responses, (b) whether chronic administration of *n*‐butylidenephthalide can regulate macrophage phenotype, and (c) whether PI3K/STAT3 axis involves the *n*‐butylidenephthalide‐mediated macrophage phenotype changes using the inhibitors of PI3K and STAT3 in a rat MI model.

## 2 METHODS

Male Wistar rats were purchased from LASCO. All experiments were conducted according to protocols approved by the China Medical University Committee on Animal Care of (permit number: 2016‐070) and conformed to the Guide for the Care and Use of Laboratory Animals published by the US National Institutes of Health (NIH Publication No 85‐23, revised 1996). Two to three rats were housed in temperature‐controlled ventilated cabinets and monitored daily for any signs of distress or clinical symptoms of illness by trained personnel. At the end of the experiment, the rats were sacrificed under sodium pentobarbitone anesthesia according to the guidelines for euthanasia.

### 2.1 Animals

#### 2.1.1 Experiment 1 (in vivo)

MI was induced by ligation of the left anterior descending coronary artery under anesthesia with ketamine‐xylazine (90 mg/kg‐9 mg/kg, intraperitoneally) in adult (2‐month‐old and 285 ± 12 g of body weight at the beginning of the study) and aged (18‐month‐old and 474 ± 14 g of body weight at the beginning of the study) male Wistar as described previously.^16^ To avoid the possibility that the measures were due to senescent decompensation, not ageing compensation, we did not use senescent rats. Twenty‐four hours after ligation, the rats were then randomly assigned into groups of either vehicle or *n*‐butylidenephthalide (150 mg/kg per day) administered orally by gastric gavage once a day. The oral dose of *n*‐butylidenephthalide was according to previous studies,^17^ showing effective attenuation of cardiovascular remodelling. The heart was excised at days 3 or 28 after MI as early and late stages of MI. The duration of the study was set at 4 weeks, as the myocardial remodelling process in rats has been reported to be mostly complete (70%‐80%) within 3 weeks.^18^, ^19^ The drug was stopped in each group approximately 24 hours before the end of the experiments to stop its pharmacological effects. Sham operation served as controls.

#### 2.1.2 Experiment 2 (ex vivo)

To investigate the effect of the PI3K/Akt signalling on *n*‐butylidenephthalide‐induced macrophage polarization after MI, infarcted rat hearts were perfused with LY294002 (a specific PI3K inhibitor) and S3I‐201 (a STAT3 inhibitor) in an ex vivo experiment. Three days after coronary ligation‐induced MI in male Wistar adult rats (2‐month‐old), the infarcted rat hearts were isolated, divided into four groups, and subjected to the following treatment: vehicle, *n*‐butylidenephthalide (15 μg/ml), or a combination of *n*‐butylidenephthalide and LY294002 (5 μmol/L, 2‐(4‐morpholinyl)‐8‐phenyl‐4*H*‐1‐benzopyran‐4‐one; Calbiochem), or *n*‐butylidenephthalide and S3I‐201 (25 μmol/L, 3‐(2,4‐dichlorophenyl)‐4‐(1‐methyl‐1H‐indol‐3‐yl)‐1Hpyrrole‐2,5‐dione; Calbiochem, La Jolla, CA, USA). The doses of *n*‐butylidenephthalide,^20^ LY294002,^21^ and and S3I‐201 22 have been shown to effectively modulate PI3K signalling. Each heart was perfused with a noncirculating modified Tyrode's solution as previously described.^16^ The drugs were perfused for 30 minutes. At the end of the study, all hearts (n = 5 each group) were measured by Western blotting for STAT3 and ELISA for PI3K activity and IL‐10 at the border zone (<2 mm outside the infarct).

### 2.2 Echocardiogram

At 28 days after operation, the rats after intraperitoneal administration of ketamine‐xylazine (45 mg/kg‐5 mg/kg) underwent echocardiographic measurements using the GE Healthcare Vivid 7 Ultra‐sound System equipped with a 14‐MHz probe as shown in the Appendix S1.

### 2.3 Haemodynamics and Infarct size measurements

Haemodynamic parameters and infarct size were measured in anesthetized rats at the end of the study. A section, taken from the equator of the LV, was fixed in 10% formalin and embedded in paraffin for determination of infarct size. The details were available as Appendix S1.

### 2.4 Flow cytometry

Quantitative analyses of LV macrophage number and phenotype were performed by flow cytometry on tissue digests at day 3 after MI. Residual blood was first rinsed from the LV which was then dissected from the border zone. Samples were digested in a mixture of collagenase IV, DNase and hyaluronidase at 37°C for 30 minutes followed by filtration through a 70 μm nylon mesh and centrifuged for 5 minutes at 600 g. Cell suspensions were washed and blocked with anti‐CD16/CD32 antibodies prior to staining. M1 macrophages were identified as CD45^+^ and CD11b^+^F4/80^+^ cells and M2 as CD45^+^ and CD206^+^ F4/80^+^ cells. Fluorescent labelling was analysed with a flow cytometer (Accuri C6; BD Biosciences).

### 2.5 Western blot analysis of STAT3, iNOS, IL‐10, and α‐SMA

Samples were obtained from either the border zone at day 3 or from the remote zone (>2 mm within the infarct) at day 28. Antibodies to phosphor (Tyr705)‐STAT3 (Cell Signaling Technology), total STAT3 (Santa Cruz Biotechnology), iNOS (Cell Signaling Technology), IL‐10 (R& D systems), α‐SMA (Clone 1A4, Sigma) and β‐actin (Santa Cruz Biotechnology) were used. Western blotting procedures were described previously.^16^ Experiments were replicated three times and results expressed as the mean value.

### 2.6 Real‐time RT‐PCR of IL‐6, IL‐1β, iNOS, CD206, and IL‐10

mRNAs were quantified by real‐time quantitative reverse transcription‐polymerase chain reaction (RT‐PCR) using TaqMan system (Prism 7700 Sequence Detection System, PE Biosystems) from samples obtained from the border zone with *cyclophilin* as a housekeeping gene at day 3 after MI. We measured the gene expression patterns for M1 (*IL‐6, IL‐1β, iNOS*) and M2 (*CD206, IL‐10*) macrophages. Sequence of PCR primers is shown in the Appendix S1.

### 2.7 Immunohistochemical analysis of STAT3, CD68, iNOS, IL‐10 and α‐SMA

In order to investigate the downstream pathway of the PI3K signalling, immunohistochemical staining was performed on LV muscle at day 3 for STAT3, CD68 (a marker for all macrophages), iNOS (a marker for M1) and IL‐10 (a marker for M2c) and at day 28 for α‐SMA (a marker for myofibroblast). Ten random scans per section were analysed and averaged. Quantification was calculated as the percentage of positively stained area to total area at a magnification of 400×. The details were available as Appendix S1.

### 2.8 Morphometry of cardiac fibrosis

For measuring cardiac fibrosis, paraffin‐embedded sections were stained with Aniline blue and picrosirius, a collagen‐specific stain (Sirius Red F3BA; Pfaltz & Bauer) from the remote zone at 28 days after MI. The density of labelled areas was qualitatively estimated from 10 randomly selected fields at a magnification of 400×. The value was expressed as the ratio of the labelled area to total area. For a detailed method, please refer to the Appendix S1.

### 2.9 Laboratory measurements

PI3K activity at the border zone was investigated using ELISA (Echelon Biosciences) according to the kit operation protocol.^23^ Each experiment was carried out as duplicate and was repeated for three times. To measure the DNA‐binding activity of STAT3, a TransAM STAT3 Transcription Factor Assay Kit (Active Motif) was used to assess the activity in myocardial homogenates according to the manufacturer's protocol. Histologic collagen analyses were further determined by the hydroxyproline assay according to Stegemann and Stalder.^24^ The samples from the remote zone were frozen immediately upon collection in liquid nitrogen and stored at −80ºC until assayed. The results were expressed as Hydroxyproline content per weight of tissue.

To measure myocardial IL‐10 activity as a biomarker of M2c, we detected myocardial membrane‐bound IL‐10 fractions using commercially available ELISA kits (R&D Systems) in myocardial homogenates from the border zones as described previously.^16^

### 2.10 Statistical analysis

All data were expressed as ± SD. Statistical analysis was conducted with the SPSS statistical package (SPSS, version 19.0). Two‐way analysis of variance was used to determine differences between conditions (sham and MI), groups (young and ageing) and intervention (vehicle and *n*‐butylidenephthalide). Post hoc pairwise comparisons were made using the Newman‐Keuls test if significant differences between the two groups were observed. A *P* value of < 0.05 was considered to indicate statistical significance.

## 3 RESULTS

### 3.1 Experiment 1 (in vivo)

#### 3.1.1 Part 1: acute stage (day 3)

Heart tissue was collected at day 3 after MI. Differences in mortality and infarct size between the young and ageing infarcted groups were not found at the acute stage of MI.

##### Effect of ageing and n‐butylidenephthalide on PI3Kand STAT3 activation

**Figure 1:**
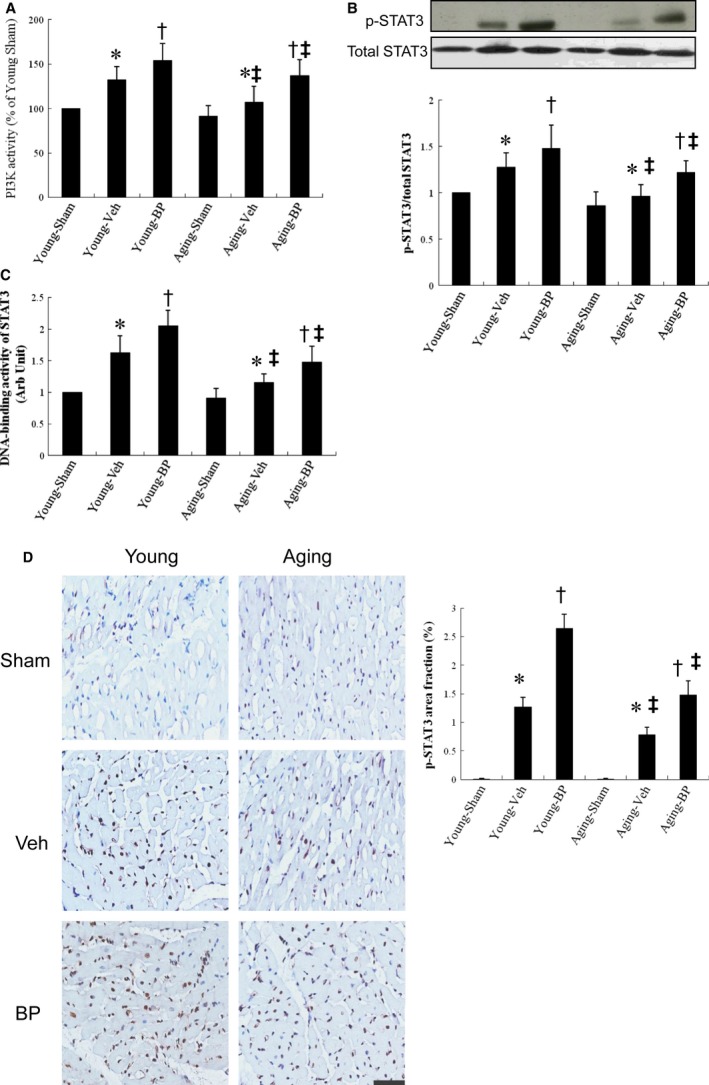
PI3K activity and STAT 3 activity analysis from the border zone at day 3 after MI. A, PI3K activity. The PI3K activity was shown as fold changes to that of the vehicle. Each experiment was repeated for three times in duplicates. B, Western analysis of STAT3. Relative abundance was obtained by normalizing the protein density against that of β‐actin. Each column and bar represents mean ± SD. Each point is an average of 3 separate experiments. C, DNA‐binding activity of STAT3 was measured by ELISA with heart homogenates. D, Representative immunostaining micrographs show p‐STAT3 nuclear translocation (brown) and quantitative analysis. The value is expressed as the ratio of p‐STAT3‐stained area to total area. *n*‐butylidenephthalide increases the nuclear translocation of STAT3 in infarcted hearts. n = 5 in each group. Bar = 50 μm. BP, *n*‐butylidenephthalide. *P < 0.05 vs sham in respective age group; ^†^ *P* < 0.05 vs vehicle in respective age group; ^‡^ *P* < 0.05 vs the same treatment in the young group

The activity of PI3K was measured by ELISA (Figure 1A). We find no difference in the PI3K activity between young and ageing sham rat hearts. However, the PI3K activity after MI was significantly upregulated compared to their respective sham, and the magnitude of increase level was lower in the ageing hearts (17% in aged and 32% in young, *P* < 0.05). Thus, although ageing hearts have similar trends in responses to injury, the responses in ageing hearts were blunted and inadequate. Besides, the PI3K activity was further increased in the *n*‐butylidenephthalide‐treated infarcted group compared with vehicle.

Western blot showed that MI increased phosphorylation of STAT3 (p‐tyr705) in both age groups (both *P* < 0.01 vs sham, respectively, Figure 1B). MI induced a 12% increase of p‐STAT3 (p‐tyr705) in the ageing rats which was significantly smaller than in young rats (12% vs 28% in the young, *P* < 0.05). *n*‐butylidenephthalide administration further increased STAT3 activation to 116 ± 12% of vehicle in young rats (*P* < 0.05) and 127 ± 15% of vehicle in ageing rats (both *P* < 0.05 vs respective vehicle).

Similarly, the DNA‐binding activity of STAT3 was significantly increased after *n*‐butylidenephthalide treatment, as measured by transcription factor ELISA (Figure 1C).

To determine the activation of myocardial STAT3, immune‐staining analysis with anti‐p‐tyr705 STAT3 antibody was performed to evaluate the nuclear translocation (Figure 1D). As a result, myocardial p‐STAT3 can be seen in the nucleus after MI. The extent of STAT3 nuclear translocation was significantly increased in the ageing infarcted rats after *n*‐butylidenephthalide administration.

##### Effect of ageing and n‐butylidenephthalide on macrophages skewing towards a M2 phenotype

**Figure 2:**
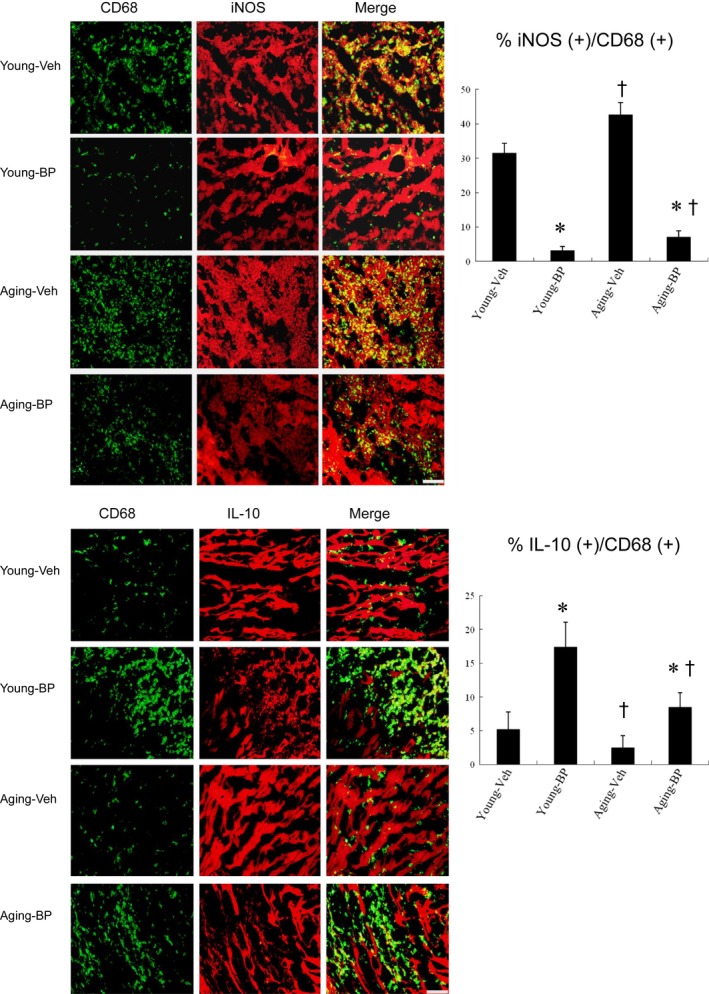
Immunohistochemical staining of M1 and M2 macrophage phenotype at the border zone at day 3 after MI. Upper panels. iNOS‐expressing CD68 (+) M1 macrophages were recruited to infarcted myocardium treated with vehicle (Veh), but were significantly reduced after *n*‐butylidenephthalide (BP) administration. The M1 ratio was significantly higher in ageing rats than in young rats. Lower panels. IL‐10‐expressing CD68 (+) M2 macrophages were predominant in BP‐administered infarcted myocardium. The ageing rats had a significant lower M2 ratio compared with the young rats. The iNOS‐expressing CD68 (+) or IL‐10‐expressing CD68 (+) macrophages were calculated and expressed as bar graphs. The values are mean ± SD of five animals from each group. The line length corresponds to 20 μm. *P < 0.05 vs vehicle in respective age group; ^†^ *P* < 0.05 vs the same treatment in the young group

Because macrophage phenotypic change is credited with an essential role in tissue repair and fibrosis, we investigated the effect of ageing and *n*‐butylidenephthalide on macrophage skewing by examining type‐specific surface markers. To determine the subtype of infiltrating macrophages, the markers for M1 (CD68^+^, iNOS^+^) and M2c (CD68^+^, IL‐10^+^) were examined in infarcted myocardium (Figure 2). Immunohistochemical staining revealed that CD68^+^ macrophages were infiltrated in the infarcted groups at day 3 after MI. Compared with young vehicle, ageing rats showed significantly higher iNOS‐expressing CD68^+^ macrophages (31.5 ± 2.8% in the young vs 42.6 ± 3.6% in the ageing, *P* < 0.05), and lower IL‐10‐expressing CD68^+^ macrophages (5.2 ± 2.6% in the young vs 2.5 ± 1.8% in the ageing, *P* < 0.05). Significantly, *n*‐butylidenephthalide administration decreased the percentage of M1 macrophages 3.1 ± 1.3% in young rats and 7.0 ± 1.8% in ageing rats (both *P* < 0.05 vs respective vehicle) and increased the percentage of M2 macrophages by 2.36‐folds in young rats and 2.41‐folds in ageing rats (both *P* < 0.05 vs respective vehicle) 3 days after MI. These data suggest improved ability of *n*‐butylidenephthalide to increase M2 macrophage differentiation with a concomitant reduction in M1.

**Figure 3:**
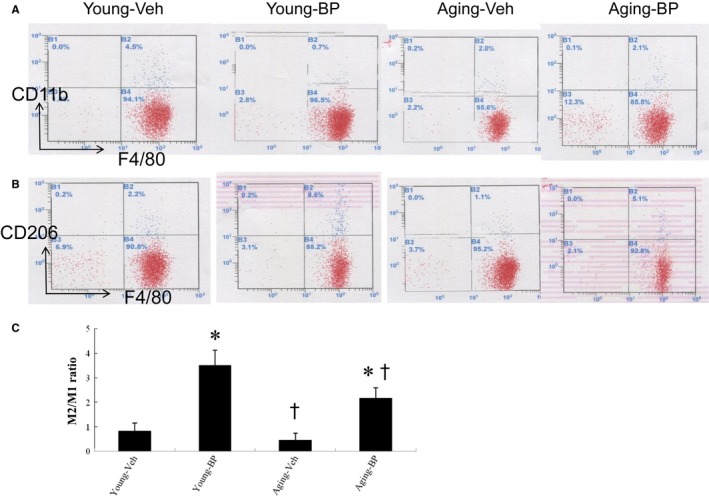
Flow cytometry analyses of macrophages at the border zone at day 3 after MI. There was macrophage accumulation at the border zone. Ageing and *n*‐butylidenephthalide (BP) induced phenotypic switch in macrophage polarization. BP significantly reduced the M1 percentage and increased the M2 percentage, resulting in increased the M2/M1 ratio. A,B: Representative flow cytometry plots showing macrophage distribution by CD11b (A) and CD206 (B). C, The ratio of M2/M1 in the myocardium. N = 5 per group. *P < 0.05 vs vehicle in respective age group; ^†^ *P* < 0.05 vs the same treatment in the young group

Besides intracellular markers for macrophage phenotype by immunohistochemical staining, we further analysed surface markers of macrophage subsets by flow cytometry. Two macrophage subsets were identified in the infarcted hearts: M1 macrophages characterized by double staining for F4/80 and CD11b, and M2 macrophages characterized by double staining for F4/80 and CD206. In the vehicle‐treated young rat hearts, M1 macrophages were the dominant population at day 3 after MI (Figure 3). *n*‐butylidenephthalide significantly increased the M2/M1 ratio compared with vehicle of respective age (both, *P* < 0.05).

**Figure 4:**
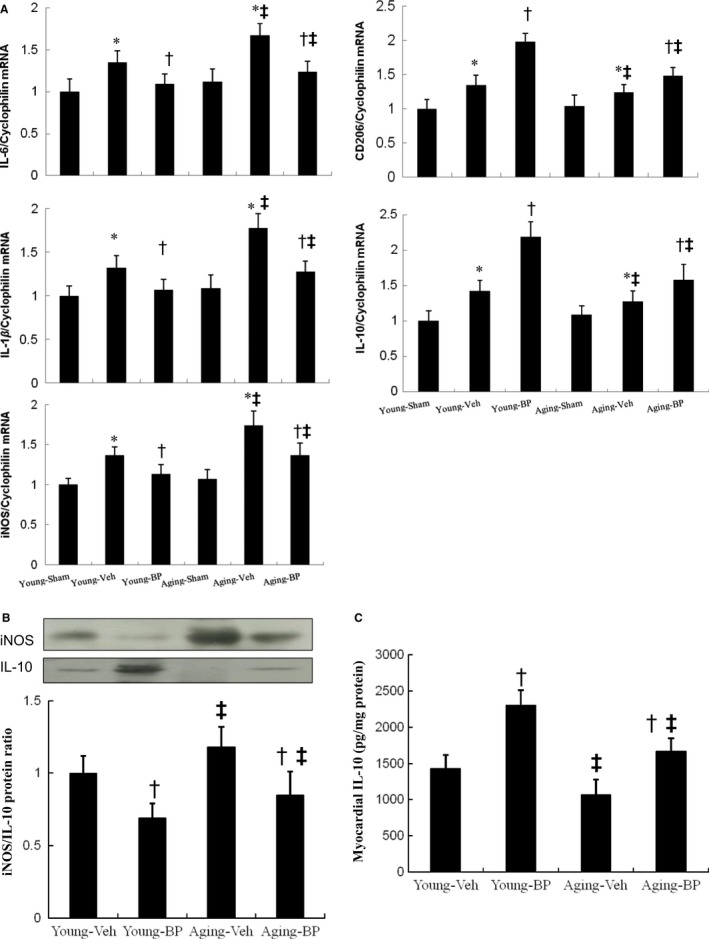
Expression of gene markers and function for M1 and M2 macrophages at day 3 after MI. A, mRNA, B, iNOS and IL‐10 protein levels and C, myocardial IL‐10 activity. There is an obvious shift towards M2 macrophage phenotype in infarcted groups treated with *n*‐butylidenephthalide (BP) as shown by changed expressions of reduced M1‐related genes (*IL‐6, IL‐1β, iNOS)* and iNOS protein levels and increased M2 genes (*CD206*, *IL‐10*) and IL‐10 protein levels. *P < 0.05 vs sham in respective age group; ^†^ *P* < 0.05 vs vehicle in respective age group; ^‡^ *P* < 0.05 vs the same treatment in the young group

Then, we analysed the expression and function of M1 and M2 macrophages. Compared with young rats, M1 (*IL‐6, IL‐1β, iNOS)* mRNA was remarkably increased and M2 *(CD206, IL‐10)* mRNA was highly deduced in the ageing rats (Figure 4A). *n*‐butylidenephthalide administration significantly decreased the M1 mRNA and increased M2 mRNA in both age groups. Then, we measured the protein levels of iNOS and IL‐10 by Western blotting (Figure 4B). Compared with vehicle, the infarcted rats treated with *n*‐butylidenephthalide had significantly lower iNOS levels and increased IL‐10 levels. The protein ratio of iNOS to IL‐10 was shown in Figure 4B. These results indicated that *n*‐butylidenephthalide administration prevents M1 polarization and enhances M2 differentiation through modulation of subtype‐specific genes and proteins.

Myocardial IL‐10 activity was presented in Figure 4C. IL‐10 activity levels were significantly higher (*P* < 0.05) in the *n*‐butylidenephthalide‐treated groups compared with vehicle in either age.

#### 3.1.2 Part 2: chronic stage (day 28)

There was similar mortality among the infarcted groups during 4 weeks after infarction. Body weight increased significantly as age increased (Table 1). LV weight in ageing rats was significantly increased compared with young rats; however, LV weights normalized by body weight at the 4th week after MI are not significant differences among the young and ageing infarcted groups. The values of ‐dP/d*t* in ageing sham were significantly decreased compared with young sham. Regardless of age, the ‐dP/d*t* data were significantly higher after administering *n*‐butylidenephthalide compared with those in the respective vehicle‐treated infarcted group. There were no significant differences in LV end‐systolic pressure, LV end‐diastolic pressure, and infarct size between the infarcted groups.

**Table 1:** Cardiac morphology, haemodynamics and echocardiographic data 4 weeks after MI

Parameters	Sham		Infarction			
	Young	Ageing	Young‐Veh	Young‐BP	Ageing‐Veh	Ageing‐BP
No. of rats	10	10	9	12	8	8
Body weight, g	352 ± 16	588 ± 18*	342 ± 16	358 ± 18	545 ± 18^‡^	564 ± 14^‡^
Heart rate, bpm	374 ± 12	348 ± 22	388 ± 15	402 ± 21	342 ± 27	352 ± 27
LVESP, mm Hg	98 ± 7	104 ± 5	94 ± 8	101 ± 7	99 ± 9	100 ± 7
LVEDP, mm Hg	6 ± 1	5 ± 3	19 ± 5	16 ± 5	20 ± 5	16 ± 5
+dP/d*t*, mm Hg/s	7071 ± 352	7342 ± 398	2873 ± 331	3376 ± 329^†^	2682 ± 372	2872 ± 307
−dP/d*t*, mm Hg/s	6872 ± 339	6092 ± 286*	2106 ± 239	2582 ± 307^†^	2017 ± 212	2431 ± 233^†^
Infarct size, %	–	–	40 ± 2	41 ± 3	41 ± 3	41 ± 2
LVW/BW, mg/g	2.22 ± 0.21	2.35 ± 0.25	3.55 ± 0.31	3.39 ± 0.38	3.69 ± 0.42	3.45 ± 0.40
LVEDD (mm)	6.1 ± 0.2	6.4 ± 0.2*	8.9 ± 0.3	7.6 ± 0.2^†^	9.6 ± 0.3^‡^	8.5 ± 0.2^‡^
LVESD (mm)	3.7 ± 0.2	4.0 ± 0.2*	7.2 ± 0.2	5.6 ± 0.3^†^	7.8 ± 0.3^‡^	6.4 ± 0.3^†^ ^,^ ^‡^
FS (%)	39 ± 3	38 ± 3	19 ± 3	26 ± 3^†^	18 ± 4	25 ± 3^†^

Note

Values are mean ± SD.

Abbreviations: BP, n*‐*butylidenephthalide; BW, body weight; LW, lung weight; LVEDD, left ventricular end‐diastolic dimension; LVEDP, left ventricular end‐diastolic pressure; LVESD, left ventricular end‐systolic dimension; LVESP, left ventricular end‐systolic pressure; LVW, left ventricular weight.

* *P* < 0.05 compared with young sham.

^†^ *P* < 0.05 vs vehicle in respective age group.

^‡^ *P* < 0.05 vs the same treatment in the young group.

#### 3.1.3 2 Echocardiography

In sham rats, LV diastolic and systolic diameters in the ageing group were significantly increased compared with the young group (Table 1). Compared with sham, MI hearts demonstrated significant increase in LV diastolic and systolic diameters, consistent with LV remodelling. Compared with young infarcted rats, ageing rats had significant increase in LVESD and LVEDD. Echocardiogram showed a significant decrease in LVESD, LVEDD and a significant increase in LV fractional shortening in *n*‐butylidenephthalide‐treated infarcted rats in comparison with vehicle‐treated infarcted rats irrespective of age.

##### Effects of ageing and n‐butylidenephthalide on myofibroblasts and cardiac fibrosis

**Figure 5:**
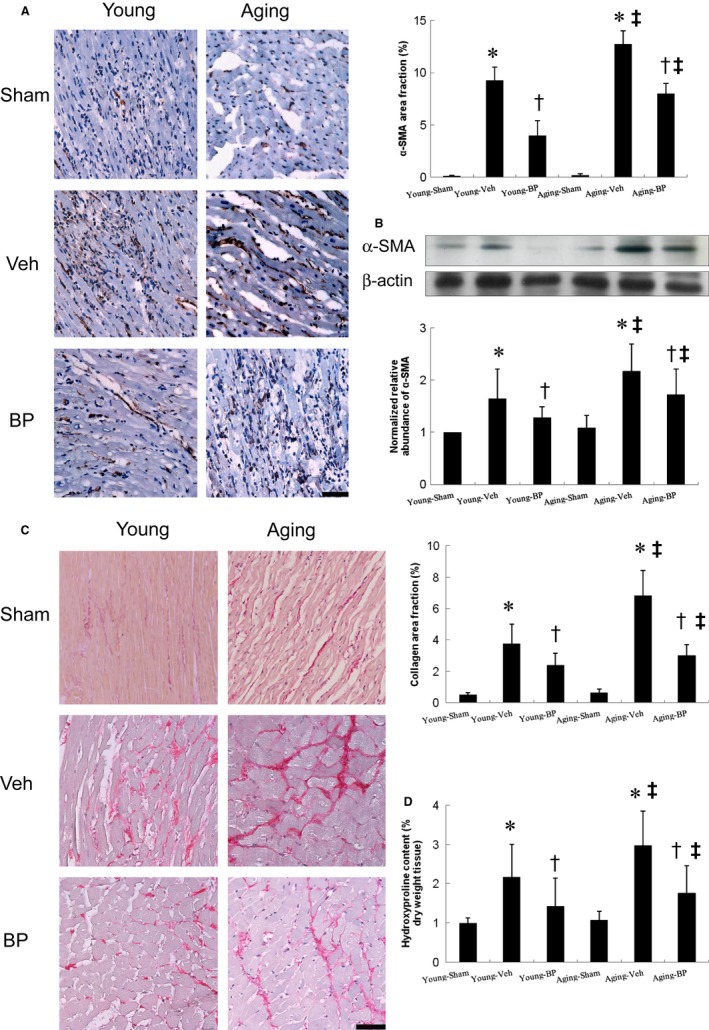
Myofibroblast infiltration and cardiac fibrosis at the remote zone at day 28 after infarction. A, Representative immunohistochemical staining of α‐SMA, a hallmark of myofibroblasts (magnification 400×). B, Western blot of α‐SMA. Western blot shows consistent results of immunohistochemical staining. C, Representative sections from the remote zone with Sirius Red staining (red, magnification 400×). D, Hydroxyproline contents to measure quantitative amount of fibrosis. The line length corresponds to 50 μm. The values are mean ± SD of 5 animals from each group. *P < 0.05 vs sham in respective age group; ^†^ *P* < 0.05 vs vehicle in respective age group; ^‡^ *P* < 0.05 vs the same treatment in the young group

α‐SMA‐expressing myofibroblasts appeared in the interstitium of the infarcted myocardium by immunohistochemistry 4 weeks after infarction (Figure 5A). The α‐SMA signals significantly increased in ageing infarcted rats were observed than in young infarcted rats. Regardless of age, *n*‐butylidenephthalide administration significantly reduced the intensity of the immunoreaction compared with respective vehicles. To further confirm the findings of immunohistochemical staining, Western blots of α‐SMA were performed. Similarly, the protein levels of α‐SMA were significantly reduced in the *n*‐butylidenephthalide‐treated groups compared with in the vehicle (Figure 5B).

Sirius Red staining to demonstrate fibrosis was examined in tissue sections of the LV from the remote zone as shown in Figure 5C. We found a slight but not significant increase of cardiac fibrosis in ageing sham rats compared with young sham rats. Ageing infarcted rats treated with vehicle had significantly larger areas of intense focal fibrosis compared with young infarcted rats (6.82 ± 1.62% vs 3.76 ± 1.24%, *P* < 0.0001). Compared with vehicle‐treated infarcted rats, treatment with *n*‐butylidenephthalide attenuated fibrosis in both age groups.

Consistently, hydroxyproline assay showed a significant increase in collagen contents after MI, and this was significantly attenuated by *n*‐butylidenephthalide (Figure 5D).

### 3.2 Experiment 2 (ex vivo)

#### 3.2.1 n‐butylidenephthalide attenuates IL‐10 level in a PI3K/STAT3‐dependent pathway

**Figure 6:**
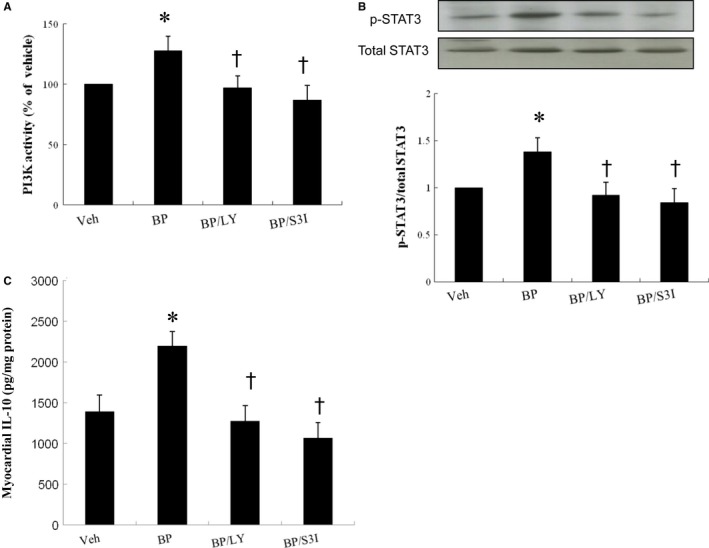
Experiment 2. (ex vivo). PI3 activity, STAT3 immunoblot and IL‐10 levels in homogenates of the LV from the border zone in an ex vivo study. In a rat isolated heart model, *n*‐butylidenephthalide significantly increased STAT3 activity and IL‐10 levels compared with vehicle, which can be abolished after administering either LY294002 (LY, a PI3K inhibitor) or S3I‐201 (an STAT3 inhibitor). Relative abundance was obtained by normalizing the density of STAT3 proteins against that of β‐actin. Results are mean ± SD of 3 independent experiments. n = 5 ineach group. *P < 0.05 vs vehicle; ^†^ *P* < 0.05 vs BP‐treated group

To investigate whether the PI3K/STAT3 signalling pathway is critical for M2c macrophage polarization, we examined the effect of LY294002 and S3I‐201 on myocardial IL‐10 levels by Western blot and ELISA (Figure 6). The requirement of PI3K in the regulation of STAT3 was shown in infarcted rats from *n*‐butylidenephthalide and from the combination of *n*‐butylidenephthalide and LY294002. LY294002 not only abolished PI3K activation but also decreased STAT3 phosphorylation. However, the STAT3‐specific inhibitor, S3I201, decreased only the phosphorylation of STAT3; there were no detectable effects on PI3K activation. These results imply that PI3K likely serves as an upstream molecule of STAT3. Furthermore, myocardial IL‐10 level markedly increased in the group treated with *n*‐butylidenephthalide alone. However, the increased IL‐10 level can be reversed after administering either LY294002 or S3I‐201.

## 4 DISCUSSION

In this study, we found that intrinsic responses to MI are unaltered but impaired with normal ageing. The findings of aged hearts impaired responses to MI were supported by a diminished injury‐induced PI3K/STAT3 activation. We found that *n*‐butylidenephthalide administration even after infarction is sufficient to rejuvenate the age‐associated impairment in macrophage polarization, mainly through activation of the PI3K/STAT3 axis. Our results were consistent with the findings of Brack et al,^25^ showing that with age, the systemic environment facilitates conversion to a fibrogenic fate. These results are consistent with the beneficial effects of *n*‐butylidenephthalide, as documented structurally by a reduction in cardiac fibrosis, increased STAT3 nuclear translocation and M2 infiltration, and attenuated myofibroblast infiltration; molecularly by myocardial STAT3, iNOS and IL‐10 protein and mRNA levels; biochemically by PI3K activity, STAT3 DNA‐binding activity, IL‐10 levels, and tissue hydroxyproline levels; and pharmacologically by antagonists. To the best of our knowledge, this finding identifies a previously unreported role of PI3K/STAT3 as molecular links mediating macrophage phenotypes of *n*‐butylidenephthalide with therapeutic potential after infarction. Indeed, our results were consistent with a recent study, showing that *Angelica Sinensis* can be used as an antiageing agent.^26^

**Figure 7:**
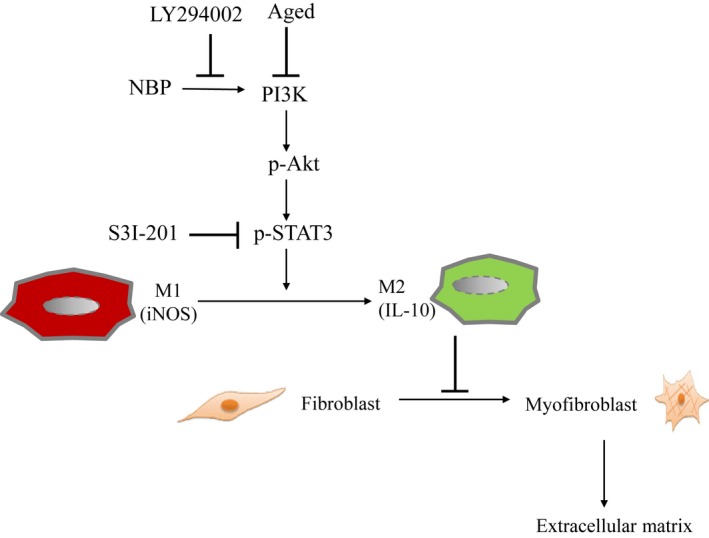
Reaction sequences illustrates the involvement of ageing and *n*‐butylidenephthalide in macrophage skewing‐ and myofibroblast‐mediated cardiac fibrosis after MI. Inflammatory M1 macrophages were dominant at the early stage of MI, however, anti‐inflammatory M2 phenotype was prevalent at a late stage. IL‐10 activity in M2 macrophages inhibits myofibroblast differentiation, which in turn reduces extracellular matrix production. *n*‐butylidenephthalide administration exhibits potent antifibrotic activity via increasing the activation of M2 macrophages and inhibiting myofibroblast differentiation. Inhibition of these signalling pathways by their respective inhibitors is indicated by the vertical lines

Our identification of the PI3K/STAT3 pathway as a necessary regulator of the macrophage phenotypes provides new insights into how fibroblasts transdifferentiated into myofibroblasts that contribute cardiac collagen deposition and fibrosis formation after MI *n*‐butylidenephthalide inhibits ageing‐ and MI‐induced activation of cardiac fibroblasts through a PI3K/STAT3‐dependent mechanism, suggesting that strategies to activate the PI3K/STAT3 pathway may represent a novel and efficacious treatment for MI. Our conclusions are supported by 3 lines of evidence (Figure 7).

### 4.1 Effect of MI on cardiac fibrosis in ageing rats

During normal ageing, age‐associated remodelling of the myocardium is characterized by the increased fibrosis, increased LV weight and dysregulated intracellular signalling when very old ageing was compared. Consistently, our results showed that with advancing age, fibrillar collagens accumulate in the LV, resulting in increased myocardial stiffness reflected by a reduction of the peak rate of the LV systolic pressure decrease (−dP/d*t* max). However, given some changes may be due to senescent decompensation, not ageing compensation, we did not use senescent rats. Thus, it is not surprising that the LV weight normalized by body weight in aged animals was similar to in young animals in this study. Indeed, our results were consistent with the observation of no differences in heart weight to body weight ratios between young and ageing hearts of 33‐month‐old.^27^

Wound healing is impaired with advancing age. During MI, ageing is associated with impaired responsiveness to injury which may be responsible for defective healing and adverse remodelling of infarcted heart. We found that despite similar infarct size, ageing‐related defects in reparative response lead to defective activation of reparative fibroblasts in response to MI. In ageing rat hearts, MI was less effective in recruiting M2 infiltration. Our results were consistent with previous findings, showing that ageing rats have more fibrosis in response to haemodynamic overload such as MI.^28^ However, the opposite results showed no age‐dependent effects on collagen deposition 7 days after MI.^29^ The discrepancy could be explained by the time factor. Given ageing is associated with an impaired reparative response to cardiac injury, ageing animals have been shown to delay collagen synthesis due to a decreased responsiveness to stimulators.^30^ Thus, it is not surprising that there were no differences in cardiac fibrosis between the young and ageing rats at the 7th day after MI; however, significant increase of cardiac fibrosis was observed in the ageing rats at 28th day after MI as shown in this study.

### 4.2 Effect of n‐butylidenephthalide on cardiac fibrosis in ageing rats

*n*‐butylidenephthalide administration leads to a relative increase in proportion of M2 macrophage population by 3 days after cardiac injury, which is earlier than the normal course. In our present study, we found that *n*‐butylidenephthalide treatment attenuated prolonged activation of pro‐inflammatory M1 macrophages at the acute stage of MI and reduced cardiac fibrosis at the chronic phase of MI. Macrophages might be targeted through the alteration of intracellular signals by drugs. The *n*‐butylidenephthalide‐induced changes in myocardial structure were manifested functionally as a smaller ventricle (LVEDD and LVESD), improved ventricular contractility (fraction shortening) and relaxation (−dP/d*t*). The improved relaxation was most likely the result of the reduced myocardial fibrosis.

### 4.3 The mechanism of n‐butylidenephthalide by which cardiac fibrosis was attenuated in ageing rats

Our analysis demonstrates that impaired responses of the senescent heart to cardiac injury are due to a failure of ageing hearts to activate an efficient transcriptional repair response. Because M2 macrophage infiltration was reduced in the aged rat heart, we analysed the STAT3 protein level in myocardium of rats aged 19 months. Previous studies have shown that STAT3 activity was reduced in ageing animals and this was associated with impaired cardioprotection conferred by ischaemic preconditioning in ageing animals.^31^ To gain a better understanding of the signalling mechanism responsible for the ageing effect on macrophage phenotypes, we examined PI3K/STAT3 axis. Aberrant activation of PI3K/STAT3 signalling is the underlying defect. In the present study, a decrease of PI3K activity and phosphorylated STAT3 levels was detected in LV proteins extracts derived from aged compared with young hearts. Treatment of infarcted hearts with *n*‐butylidenephthalide upregulates PI3K and STAT3 activity and IL‐10 levels in in vivo and ex vivo studies. In addition, we demonstrated that pharmacological inactivation of PI3K and STAT3 abolished myocardial IL‐10 levels. Because LY294002 inhibits PI3K and thereby not only STAT3 but also other downstream proteins kinases (for example, mitogen‐activated protein kinases,^32^ this may help to explain its varying effect on IL‐10 per se. However, and most importantly, the effect of *n*‐butylidenephthalide on IL‐10 levels was abolished in S3I‐201‐treated hearts, demonstrating the importance of STAT3 phosphorylation for IL‐10. These data confirm and extend our recent findings suggesting that M2 polarization is required for attenuated collagen production through a STAT3‐mediated pathway.

M2c macrophages inactivate myofibroblasts through the local production of IL‐10.^33^ Once macrophages adhere to cardiac fibroblasts, it is highly likely that the microenvironment produced by macrophages will affect fibroblast behaviour. In fact, recent work has demonstrated that M2 macrophages can inhibit fibroblast‐to‐myofibroblast differentiation via an IL‐10‐mediated mechanism.^33^

## 5 CLINICAL IMPLICATIONS

Given the ageing population dramatically increases, the need for treatment strategies for the aged is more important than ever before. Yet, the vast majority of research is performed in young animals. The question of whether cardioprotective responses are maintained in the ageing is unknown. This issue is crucial because the elderly population is well recognized to be the specific subset in which acute MI is most frequent. Ageing is an important factor determining cardiovascular morbidity and mortality in human population.^34^ Our study showed that older animals fare worse following MI than their younger counterparts. This age‐associated impairment in repair potential may contribute to the suboptimal clinical outcomes reported after MI in elderly individuals. This age‐related poor prognosis is able to be ameliorated with pharmacologic therapies. Pro‐inflammatory M1 macrophage activation contributes crucially to the progression of age‐associated diseases in the elderly. *n*‐butylidenephthalide attenuated the negative effects of pro‐inflammatory M1 macrophages that build up in ageing animals. Alteration in endogenous cardioprotective signalling by post‐infarct remodelling is not a negligible problem in the clinical arena. This study may offer a foundation to develop novel therapies for the treatment of cardiovascular disease associated with ageing.

## 6 STUDY LIMITATIONS

Inspite of the PI3K/STAT3 axis as a cell‐autonomous mechanism to mediate macrophage polarization in this study, we did not exclude the possibility of tissue microenvironmental cells to contribute to the activation of inflammatory signalling in aged individuals. Given our in vivo model does not dissect the role of other cells present in the heart, such as cardiomyocytes, lymphocytes and neutrophils which may influence macrophage and fibroblast function, we did not explore the possibility that the observed effects of M2 macrophage infiltration were through direct effects of *n*‐butylidenephthalide on cardiomyocytes or cell types other than via cardiac macrophage activity. Furthermore, cardiac fibroblasts have been shown to affect the macrophage phenotypes by secreting cytokines.^35^ Because macrophages are vulnerable to environmental cues, it will be important to examine the interplay at the cellular level between autonomous and nonautonomous mechanisms to elucidate age‐associated changes in macrophages. Second, given the degradation of collagen fibres takes place after a half‐life of 80‐120 days 36 and myofibroblasts persist for a long time in the heart,^37^ it is unlikely that the attenuated myofibroblast infiltration after adding *n*‐butylidenephthalide is due to the myofibroblast senescence. Finally, it is possible that, with a longer follow‐up than that of the current study, LV decompensation associated with an increased mortality rate would have been observed in the elderly cohort after MI.

## 7 CONCLUSIONS

Our study shows for the first time that *n*‐butylidenephthalide may have the potential as a therapeutic agent for MI via promotion of M2 macrophage polarization. Our study demonstrates that *n*‐butylidenephthalide protects against myocardial fibrosis in infarcted rats for targeting the PI3K/STAT3 signalling pathway. We suggest that drug‐based therapies founded on manipulating M2 polarization represent complementary strategies.

## CONFLICT OF INTEREST

The authors confirm that there are no conflicts of interest.

## AUTHORS' CONTRIBUTIONS

CCL and TML performed the research; CCL SYC and HYL designed the research study; TML and SZL analysed the data; CCL wrote the paper; All co‐authors revised the manuscript and approved the final submitted version.

## Data Availability

The data that support the findings of this study are available from the corresponding author upon reasonable request.
