# Supplementary material for: Targeting the PI3K/STAT3 axis modulates age‐related differences in macrophage phenotype in rats with myocardial infarction
Source: J Cell Mol Med. 2019 Jul 17;23(9):6378–92. doi: 10.1111/jcmm.14526 (PMC6714172; doi:10.1111/jcmm.14526)
Supplement: Supplementary file 1 [file JCMM-23-6378-s001.docx]

**Appendix S1**

***Echocardiogram***

At 28 days after operation, rats were lightly anesthetized with intraperitoneal injection of ketamine-xylazine (45 mg/kg-5 mg/kg). Echocardiographic measurements were done using the GE Healthcare Vivid 7 Ultra-sound System (Milwaukee, WI) equipped with a 14-MHz probe as described previously^1^. M-mode tracing of the LV was obtained from the parasternal long-axis view to measure LV end-diastolic diameter dimension (LVEDD), LV end-systolic diameter (LVESD) and fractional shortening (FS, %). After this, the rats quickly underwent hemodynamic measurement after systemic heparinization.

##### *Hemodynamics and infarct size measurements*

Hemodynamic parameters were measured in anesthetized rats with ketamine-xylazine (90 mg/kg-9 mg/kg) intraperitoneally at the end of the study. A polyethylene Millar catheter was inserted into the LV and connected to a transducer (Model SPR-407, Miller Instruments, Houston, TX) to measure LV systolic and diastolic pressure as the mean of measurements of five consecutive pressure cycles as previously described^1^. The maximal rate of LV pressure rise (+dP/d*t*) and decrease (-dP/d*t*) was measured. After the arterial pressure measurement, the atria and the right ventricle were trimmed off, and the LV was rinsed in cold physiological saline, weighed, and immediately frozen in liquid nitrogen after obtaining a coronal section of the LV for infarct size estimation. A section, taken from the equator of the LV, was fixed in 10% formalin and embedded in paraffin for determination of infarct size. Each section was stained with hematoxylin and eosin, and trichrome. The infarct size was determined as previously described^2^.

***Real-time RT-PCR of IL-6, IL-1β, iNOS, CD206, and IL-10***

Real-time quantitative RT-PCR was performed from samples obtained from the border zone with the TaqMan system (Prism 7700 Sequence Detection System, PE Biosystems) at day 3 as previously described^1^. We analyzed the expression of gene markers for M1 (*IL-6, IL-1β, iNOS*) and M2 (*CD206, IL-10*) macrophages. Primers sequences were the following:

*IL-6* sense 5'-CCAGTTGCCTTCTTGGGACTGATG-3', antisense 5'-ATTTTCTGACCACAGTGAGGAATG-3';

*IL-1β* sense 5'-ATGGCAACTGTCCCTGAACTCAACT-3', antisense 5'-CAGGACAGGTATAGATTCAACCCCTT-3';

*iNOS* sense 5'-TCACCTTCGAGGGCAGCCGA-3', antisense 5'-TCCGTGGCAAAGCGAGCCAG-3';

*CD206* sense 5'-TGGGTTTGCTGAAGAAGAGAA-3', antisense 5'-CATGTGATAAGTGACAAATGCTTG-3';

*IL-10* sense 5'-GGTTGCCAAGCCTTGTCAGAA-3', antisense 5'-GCTCCACTGCCTTGCTTTTATT-3';

*cyclophilin* sense 5’-ATGGTCAACCCCACCGTGTTCTTCG-3’, antisense 5’-CGTGTGAAGTCACCACCCTGACACA-3’.

Standard curves were plotted with the threshold cycles versus log template quantities. After initial denaturation, amplification was performed at 95°C (10 s) 60°C (5 s) 72°C (10 s) for 45 cycles. Fold change was normalized against *cyclophilin*, a housekeeping gene.

***Immunohistochemical analysis of STAT3, CD68, iNOS, IL-10 and α-SMA***

To confirm the downstream pathway of the PI3K signaling, immunohistochemical staining was performed on LV muscle at day 3 for STAT3, CD68, iNOS and IL-10 and at day 28 for α-SMA. Cryosections incubated with antibodies against phospho(Tyr705)-STAT3 (Cell Signaling Technology, Danvers, MA, USA), CD68 (a marker for all macrophages; Abcam, Cambridge, MA), iNOS (a marker for M1; Cell Signaling Technology, Danvers, MA, USA), IL-10 (a marker for M2c; R& D systems, Abingdon, UK), and α-SMA (a marker for myofibroblast; Sigma, St. Louis, Missouri). The antibody had been tested for specificity in the rat. Isotype-identical directly conjugated antibodies served as a negative control. Ten random scans per section were analyzed and averaged. Quantification was calculated as the percentage of positively stained area to total area at a magnification of 400×.

***Morphometry of cardiac fibrosis***

Aniline blue and picrosirius staining, a collagen-specific stain (Sirius Red F3BA; Pfaltz & Bauer, Stamford, CT), were used to stain 5-µm thick, paraffin-embedded sections in coronal sections of the remote zone at 28 days after MI. The interstitial collagen fraction was determined by quantitative morphometry of the picrosirius-stained sections with an automated image analyzer (Image Pro Plus, CA). These parameters were assessed in a blinded fashion by at least two investigators. The density of labeled areas was qualitatively estimated from 10 randomly selected fields at a magnification of 400×. The value was expressed as the ratio of the labeled area to total area.

***Laboratory measurements***

PI3K activity at the border zone was measured using a commercial ELISA kit (Echelon Biosciences, Salt Lake City, UT, USA) according to the manufacturer’s instructions^3^. In this method, PI3K activity was assessed by detecting the conversion of PI(4,5)P2 into PI(3,4,5)P3. Each experiment was repeated for three times in duplicates. To evaluate the DNA-binding activity of STAT3, myocardial homogenates were prepared and a TransAM STAT3 Transcription Factor Assay Kit (Active Motif) was used according to the manufacturer’s protocol. Histologic collagen results were confirmed by hydroxyproline assay adapted from Stegemann and Stalder^4^. The samples from the remote zone were immediately placed in liquid nitrogen and stored at -80ºC until measurement of the hydroxyproline content. The results were calculated as hydroxyproline content per weight of tissue.

Myocardial IL-10 activity was assayed for M2c*.* Myocardial tissues from the border zones were homogenized in extraction buffer (50 mM potassium phosphate buffer, pH 7.0; 1 mM EDTA; 1 mM ethylene glycol tetraacetic acid; 0.2 mM phenylmethanesulfonylfluoride; 1 μg/mL pepstatin; 0.5 μg/mL leupeptin; 10 mM NaF; 2 mM Na_3_VO_4_; and 10 mM β-mercaptoethanol), and centrifuged for 30 minutes at 14,000*g* at 4°C. Myocardial membrane-bound IL-10 fractions were measured using commercially available ELISA kits (R&D Systems).

**References**

1. Lee TM, Lin SZ, Chang NC. Nicorandil Regulates the Macrophage Skewing and Ameliorates Myofibroblasts by Inhibition of RhoA/Rho-kinase Signaling in Infarcted Rats. *J Cell Mol Med* 2018;**22**:1056-69.

2. Pfeffer MA, Braunwald E. Ventricular remodeling after myocardial infarction. *Circulation* 1990; **81**:161-72.

3. Cao CM, Zhang Y, Weisleder N, Ferrante C, Wang X, et al. MG53 constitutes a primary determinant of cardiac ischemic preconditioning. *Circulation* 2010;**121**: 2565–74.

4. Stegemann H, Stalder K K. Determination of Hydroxyproline. Clin Chim Acta 1967;18: 267–73.
